# Frailty Level Monitoring and Analysis after a Pilot Six-Week Randomized Controlled Clinical Trial Using the FRED Exergame Including Biofeedback Supervision in an Elderly Day Care Centre

**DOI:** 10.3390/ijerph16050729

**Published:** 2019-02-28

**Authors:** Iranzu Mugueta-Aguinaga, Begonya Garcia-Zapirain

**Affiliations:** 1 Rehabilitation Service, Cruces University Hospital, Plaza Cruces s/n, 48903 Barakaldo, Spain; 2 eVIDA Laboratory, University of Deusto, Avda Universidades 24, 48007 Bilbao, Spain; mbgarciazapi@deusto.es

**Keywords:** frailty, elderly people, exergame, physical activity, kinect, biofeedback

## Abstract

*Background*: Frailty is a status of extreme vulnerability to endogenous and exogenous stressors exposing the individual to a higher risk of negative health-related outcomes. Exercise using interactive videos, known as exergames, is being increasingly used to increase physical activity by improving health and the physical function in elderly adults. The purpose of this study is to ascertain the reduction in the degree of frailty, the degree of independence in activities of daily living, the perception of one’s state of health, safety and cardiac healthiness by the exercise done using FRED over a 6-week period in elderly day care centre. *Material and Methods*: Frail volunteers >65 years of age, with a score of <10 points (SPPB), took part in the study. A study group and a control group of 20 participants respectively were obtained. Following randomisation, the study group (20) took part in 18 sessions in total over 6 months, and biofeedback was recorded in each session. *Results*: After 6 weeks, 100% of patients from the control group continued evidencing frailty risk, whereas only 5% of patients from the study group did so, with *p* < 0.001 statistical significance. In the case of the EQ-VAS, the control group worsened (−12.63 points) whereas the study group improved (12.05 points). The Barthel Index showed an improvement in the study group after 6 weeks, with statistically significant evidence and a value of *p* < 0.003906. Safety compliance with the physical activity exceeded 87% and even improved as the days went by. *Discussion*: Our results stand out from those obtained by other authors in that FRED is an ad hoc-designed exergame, significantly reduced the presence and severity of frailty in a sample of sedentary elders, thus potentially modifying their risk profile. It in turn improves the degree of independence in activities of daily living and the perception of one’s state of health, proving to be a safe and cardiac healthy exercise. *Conclusions*: The study undertaken confirms the fact that the FRED game proves to be a valid technological solution for reducing frailty risk. Based on the study conducted, the exergame may be considered an effective, safe and entertaining alternative.

## 1. Background

Frailty is a state of pre-disability, where there is a risk of developing a new disability from a situation of emerging functional limitation. Its importance comes from the fact that it focuses on the functionality and not on the diagnosis of disease [1]. Frailty refers to a state of extreme vulnerability in terms of both endogenous and exogenous stressors that may result in the individual being exposed to a greater risk of negative health-related situations [2]. Frailty may represent a transition phase between successful aging and disability, and a condition to target for restoring robustness in the individual at risk. An all-encompassing approach is needed to target frailty, bearing in mind it has the features of a syndrome. The identification of frailty as a target for implementing preventive interventions against age-related conditions is pivotal. Every effort should be made by health care authorities to maximize efforts in this field, balancing priorities, needs, and resources. It is important to raise awareness about frailty and age-related conditions among the population at large so as to ensure effective prevention, with a view to promoting healthy types of behaviour and lifestyles [3,4,5,6].

During the past decade there has been a rapid increase in research into the use of technology in the elderly population [7,8,9]. Exercise using interactive video games, known as exergames, is being increasingly used to increase physical activity by improving health and the physical function in elderly adults [7,10,11,12]. There is growing interest in the use of exergames as a potential rehabilitation tool to facilitate physical exercises in different clinical groups [13,14]. 

A previous pilot three-week randomised controlled trial was realised in which exclusively, FRED game was checked to improve the degree of frailty [15]. The aim of this article was to perform a six-week randomised controlled trial in the course of which monitoring was carried out over 6 weeks using the FRED game, designed under the exergame concept to ascertain any reduction in the degree of frailty. The degree of independence in activities of daily living was also analysed, as was the perception of one’s state of health and quality of life. Physiological parameters were also recorded such as the following: systolic blood pressure, diastolic blood pressure, heart rate and blood oxygen saturation in order to assess the safety and cardiac healthiness of the exercises done using FRED.

## 2. Material and Methods

### 2.1. Participants and Simple Size

Two elderly day control centers were contacted and the necessary authorisation requested and granted by the management at these centers. Approval was also requested and granted by the Deusto Foundation Research Ethics Committee. Approval was requested and obtained from the Ethics Committee in Research of the Deusto Foundation (University of Deusto), reference number: ETK-17/15-16, in order to proceed with the study. After receiving all relevant information, all the participants signed an informed consent. The consent of publication was given by all participants. The study adheres to CONSORT guidelines. Raw data are anonymously stored according to the Organic Law 15/1999 of 13 December on the Protection of Personal Data (BOE 298, 14 December 1999) and the ethical guidelines of good scientific and clinical practice. Saving the data base at a repository is making progress. Trial Registration: The protocol has been registered under ClinicalTrials.gov with the identifier: NCT03425227.

Interested parties were recruited from both day care centers via informative talks, these talks being open to all day care center participants and advertised via posters and pamphlets. Interested participants signed the duly-informed consent form. Screening was arranged for 65 participants in total in accordance with the following criteria:

*Inclusion criteria*: persons over 65 years of age with a Barthel score equal to or above 90 points who carry out no scheduled physical activity.
*Exclusion criteria*: persons over 65 years of age with a Barthel score less than 90 points or with a Barthel score equal to or above 90 points who carry out scheduled physical activity.

Of the 65 individuals who showed interest in taking part in the study, 46 met the inclusion criteria, and these participants were again asked to carry out some specific tests in order to evidence their degree of frailty. Regarding the calculation of the sample size, the required size was 19 for each group. This sample size was calculated considering the following standard values: significant level (95%) and power (80%). At the same time the a priori estimated proportions were 10% for the control group and 50% for the study group according to clinical criteria.

The sample size was calculated using the formulas corresponding to the comparison of two proportions [16]. As the minimum calculated number of participants was achieved, recruitment was not continued.

### 2.2. Experimental Design

A pilot six-week randomised controlled trial was carried out introducing biofeedback with physiological constants as follows: systolic blood pressure, diastolic blood pressure, heart rate and blood oxygen saturation in the intervention group. The main objective of the experiment was to reduce the degree of frailty evidenced in the participants, with secondary objectives being taken into consideration as follows: on the one hand, to observe whether the degree of independence and quality of life improved, and ascertain whether there was greater involvement in, adherence to and compliance with the exercise; on the other, to note whether the exercise carried out lies within certain cardiac healthy and safe parameters. 

#### 2.2.1. Section of Tools

The following scales were used in this study, and these were authorised for use by different authors following prior request for permission:

*Barthel Index:* a scale with which a quantitative estimate is obtained of the degree of independence a participant has to pursue activities of daily living. The score ranges 0-100, where 0 means total dependence and 100 means independence. [17,18]:
*EuroQol 5D-5L:* a standardised measure for state of health developed by the EuroQol Group in order to provide a single, general measurement applicable to a wide range of health conditions and treatments, which in turn provides a single descriptive profile and a unique state of health rate that may be used in clinical assessment. The individual themselves assesses their state of health―first in terms of seriousness via dimensions (descriptive system) and then via a visual analogue scale (VAS) for more general assessment purposes [19,20].
*The System Usability Scale (SUS):* this is a questionnaire based on the Likert scale to rate the capacity for using systems. The score may be between 0 and 100, in which 100 represents the best usability and a score of ≥68 is considered positive [21].
*The Short Physical Performance Battery (SPPB):* This battery includes three tests: balance test, gait speed and standing up from/sitting down on a chair five times. These tests follow a hierarchical sequence. In the balance test, the participant tries to maintain three positions: side-by-side stand, semi-tandem and tandem stand for 10 seconds each. In the gait speed test, the participant walks a distance of 4 metres at their normal pace, this test is performed twice and the shortest time is recorded. Lastly, in the case of the chair stand test, the participant stands up from and sits down on a chair 5 times as quickly as possible, and the total time taken is recorded. Each test is given a score from 0 (worst performance) to 4 (best performance): for the balance test and according to a hierarchical combination of performance in the 3 component subtests and the other 2 tests, a 0 score is assigned to those that do not complete or try out the test, and scores from 1 to 4 according to the time taken. A total score is obtained for the whole battery, which is the sum of that obtained from the 3 tests and ranges between 0 and 12, with scores below 10 evidencing frailty [1,22,23,24].
The short physical performance battery (SPPB) was used for frailty screening, this being validated and normalised for use within our working environment, and combines balance testing, gait speed and chair stand. Prioritization was based on the fact that successful validation had been hitherto successfully undertaken using this tool to detect frailty and that it ensures reliability in predicting disability [1,22,23].

#### 2.2.2. Intervention

Of the 46 participants, 40 obtained scores below 10, i.e. these 40 participants may be considered frail. In addition to the Barthel scale, the 40 participants filled in the following questionnaire: EuroQol 5D-5L. Medical records related to risk of heart failure were also gathered. 

Randomisation was carried out by classifying the 40 participants according to range of age, gender and Barthel score. A study group and a control group of 20 participants respectively were obtained. 

Participants belonging to the study group underwent three sessions per week over a 6-week period, with each session consisting of 20 minutes’ activity divided into three parts. The first targeted both the upper and lower extremities, while the second and third targeted the upper and lower extremities respectively. After completing each part, the participant was then able to assess the exertion made by means of the simplified Borg scale [25]. Depending on how they rated this, participants would either be in a position to proceed immediately, or after having done some abdominal-diaphragmatic breathing exercises of varying duration, depending on how they rated the initial exertion made. Following completion of these breathing exercises, participants would then either opt to proceed with the activity or not. The intervention sessions were supervised.

*The physiological constants—blood pressure, heart rate and blood oxygen saturation:* were recorded prior to commencing the FRED game, immediately after completing it and after 5 minutes had elapsed, according to the publications reviewed [26,27,28].

**Figure 1:**
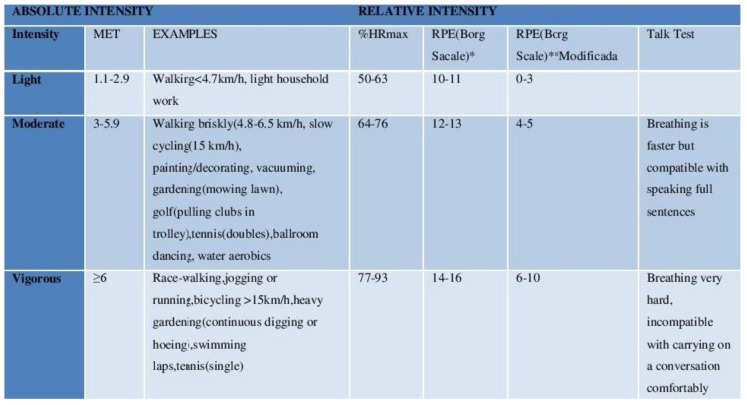
Classification of intensity of physical activity. Examples of absolute and relative levels of intensity. Source: Modified from Howley [29]. MET= metabolic equivalent); RPE = rating of perceived exertion) (*20 value Borg score) (**10 value Borg score). % HRmax = percentage of measured or estimated maximum heart rate (220-age). Heart rate (HR): <76% Maximum heart rate (%HRMAX)(*); Systolic blood pressure (SBP): <150 mmHg; Diastolic blood pressure (DBP): <90 mmHg; Blood oxygen saturation (SpO2): Variations <5%SpO2. (*)Modified Borg scale: a 5 score on the Borg scale is included together with the maximum heart rate percentage in order to ascertain that the perceived exertion is correctly related to heart rate.

The following parameters were established taking into account these publications [26,27,28], above all to assess the fact that under no circumstances may the pursuit of any physical exercise using the FRED game put the participant at any risk, thus ensuring that the activity is safe and, in turn, to assess whether it may also be beneficial to cardiovascular health (Figure 1): 

After completing the FRED game session each day, each participant from the study group was asked 2 simple questions, with just a YES or NO answer:

“Do you like the game?” and “Do you find it motivating for the purpose of improving your physical condition?”.

The participants belonging to the control group continued to lead their daily lives in the course of which they had no physical activity scheduled. After 6 weeks and having taken part in 18 physical activity sessions with the FRED game, the short physical performance battery (SPPB) test was once again carried out to ascertain whether the degree of frailty had been reduced. The Barthel score and the EuroQol 5D-L questionnaire were in turn run passed the participants and lastly, the system usability scale (SUS) was applied exclusively to participants from the intervention group.

#### 2.2.3. The Game

The FRED game was designed to be used a type of exergame [30], in which the first author of this article, in the course of their work as a physiotherapist, created its contents while taking into consideration both specific movements for developing physical exercise and the need to devise different scenarios in which such movements might be developed [29]. These scenarios evolve in a logical order to ensure that the individuals who pursue them may find the activity beneficial. Each movement is designed by taking into account both biomechanical and neuromotor parameters, and offer sufficiently wide-ranging features to enable them to be recognised by the Kinect sensor. FRED has been developed using a 3D unity motor, whereby a Kinect game controller needs to be connected to a computer and screen or TV. 

Description of the game: the game unfolds on an estate where there is a road lined with tree that leads to a farmhouse with a vegetable garden and vineyards. The game entails several scenarios, with each one representing one or more steps in a simplified process to enable txakoli to be produced [31]. The user starts performing the different activities in order and operated by remote control, with each activity referring to a specific movement of the upper and/or lower extremity. 

Different previously-defined movements are worked on that the Kinect sensor detects in order to ensure that the exercises have been suitably done. Each movement has a specific time range in which it is recorded, giving a positive score to successfully-completed exercises and showing the final score at the end of each part.

**Figure 2:**
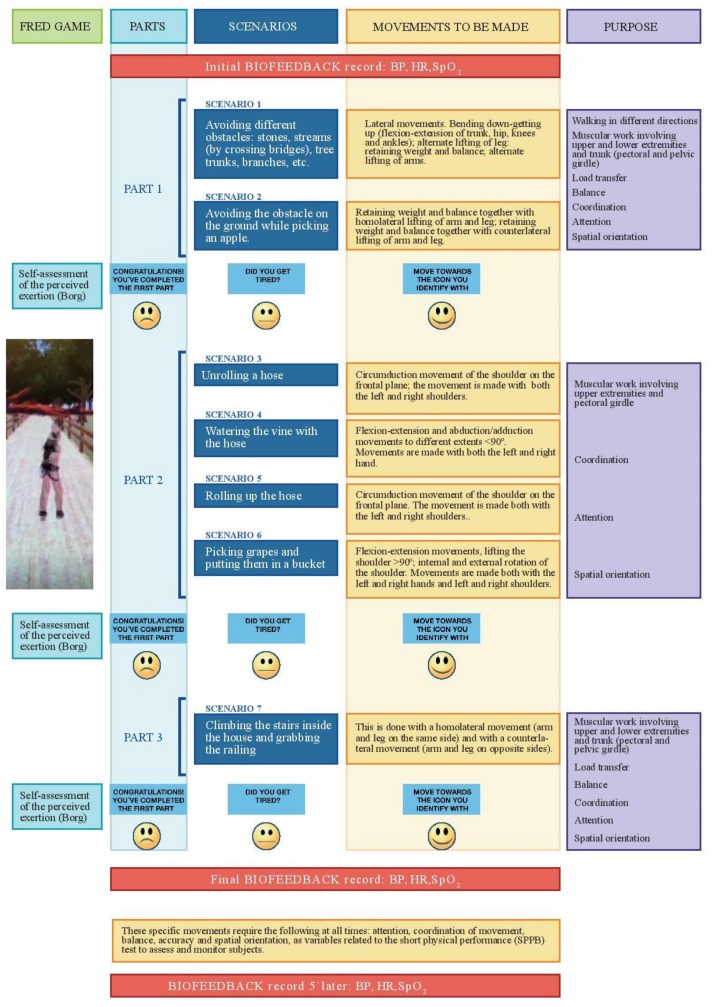
FRED game overview: structure and contents.

Below the different scenarios of the game with be considered and in sequential order. It is described every single movement that is necessary to carry out, what is their objective and why they have been used (Figure 2).

### 2.3. Statistical analysis

The R open code statistical programme version 3.2 for Windows was used to conduct the statistical tests and create the graphs. The Wilcoxon Exact Test was used to compare the average scores obtained by the study group both prior to undertaking the test and 6 weeks afterwards. The ratio between improvement in their results and the study group to which the participant belonged was calculated using Pearson and Fisher-Exact tests. An adjusted Cochran-Mantel-Haenszel estimate was used to compare the relative risks involved when classifying data according to age and gender. 

## 3. Results

### 3.1. Description of the Process

**Figure 3:**
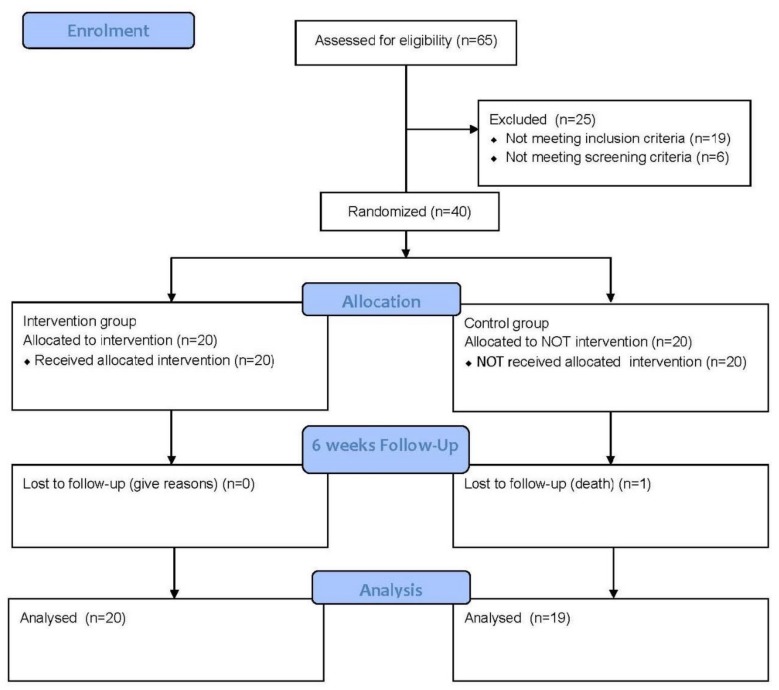
CONSORT Flow diagram of the progress through the phases of a parallel randomised trial of two groups [32].

The following CONSORT flow chart [32] outlines the complete procedure that took place in the course of the study (Figure 3). Therefore, of the 40 participants who commenced the study, 39 completed it. One participant from the control group passed away before the study was completed.

### 3.2. Description of the Sample in Week 1

**Figure 4:**
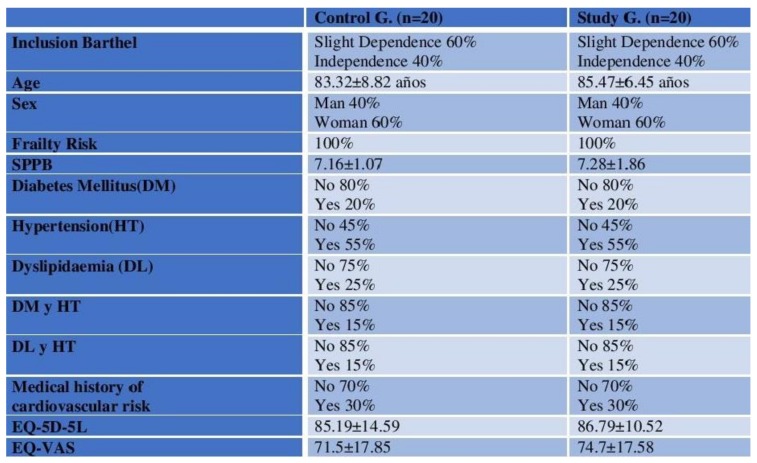
Description of sample features. DM: Diabetes Mellitus; HT: Hypertension; DL: Dyslipidaemia; EQ 5D-5L y EQVAS.

**Figure 5:**
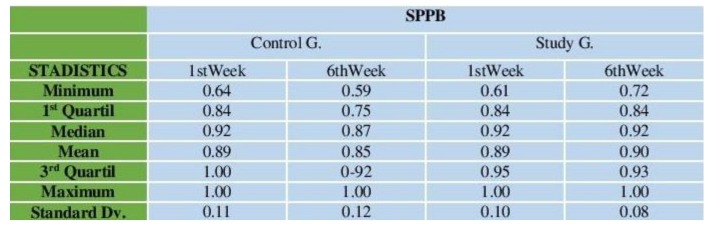
Statistical description of SPPB results in the first and sixth weeks of the study.

The sample showed to be homogeneous in terms of the Barthel Index, age, gender and frailty risk. The following table summarises the major features of both groups (Figure 4). Both groups were noted as being 100% at risk of showing signs of frailty during the first week of study, and it was also noted that both average scores were similar even though the control group showed greater minimum and maximum ranges than the study group. (Figure 5). 

### 3.3. Results Obtained from the Short Physical Performance Battery (SPPB) after 6 Weeks

**Figure 6:**
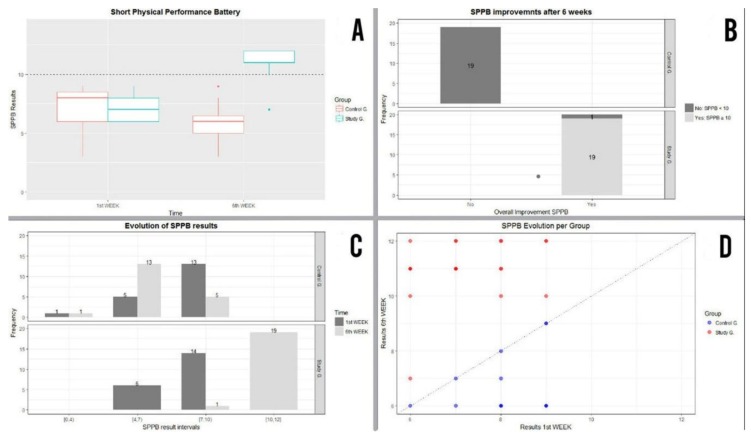
(**A**) Score obtained in the SPPB in week 1 and week 6. (**B**) Percentage frailty and number of participants in control and study group at the end of week 6. (**C**) SPPB score evolution. (**D**) Distribution of SPPB score obtained according to age and gender.

Following 6 weeks and after 18 physical activity sessions had been undertaken using the FRED game and once the results obtained from the tests had been analysed using the SPPB, the results obtained by the study group were seen to have substantially improved while, conversely, those obtained by the control group had worsened, as shown in Figure 5. After 6 weeks, all patients from the study group except for 1 obtained a score over 10, i.e., they were no longer considered to be exposed to frailty risk in accordance with the SPPB tests (Figure 6A). 

Conversely, all patients from the control group remained exposed to frailty risk (with a score below 10) after 6 weeks. The Wilcoxon Test was used to show whether results from the study group had improved after 6 weeks, with this test rejecting the initial hypothesis put forward that considered the scores obtained before and after the test to be similar (namely, *p* < 0.001). 

By the sixth week, 100% of patients belonging to the control group were seen to continue showing signs of frailty risk, whereas only 5% of patients from the study group evidenced frailty risk. No patient from the control group showed any improvement (increase in score) in their SPPB results, whereas 100% of patients from the study group showed improvements in their results after 6 weeks (Figure 6B). 

By applying the Fisher-Exact test on this information, strong evidence was obtained to suggest that there was a difference in the proportion of patients who improved their SPPB in general between the two groups (study and control), with statistical significance of *p* < 0.001. The scores obtained by participants who undertook SPPB tests during the first week and following 6 weeks are shown below, according to category (Figure 6C

As regards evolution of SPPB results and bearing in mind the intervals shown, eight participants from the control group were seen to have fallen from interval (7,10) to the interval immediately below, whereas all the participants from interval (4,7) in the study group moved up to intervals (10,12). Moreover, 13 participants moved up from interval (7,10) to interval (10,12), while one patient remained at interval (7,10), as their SPPB score 9 failed to change after 6 weeks. 

SPPB scores regarding age and gender did not need to be compared because the effect of these variables was minimised by dint of there being a homogenous sample of participants within the control and study groups (Figure 6D).

On the one hand, by using the Fisher-Exact test strong evidence was found to suggest that there was no difference in the proportion of male and female patients without risk of being exposed to frailty (SPPB ≥ 10) after 6 weeks, with statistical significance of *p* < 0.001.

And on the other, the Fisher-Exact test was adjusted according to age (85 years and over). Once again, strong evidence was found to suggest that there was no difference in the proportion of patients of 85 years and over and those under 85 years of age without risk of being exposed to frailty (SPPB ≥ 10) after 6 weeks, with statistical significance of *p* < 0.001. Both results were obtained as of the homogeneity existing between the control group and the study group in terms of age and gender (Figure 6D).

### 3.4. 5D-5L

#### 3.4.1. EQ-5D-5L Index

**Figure 7:**
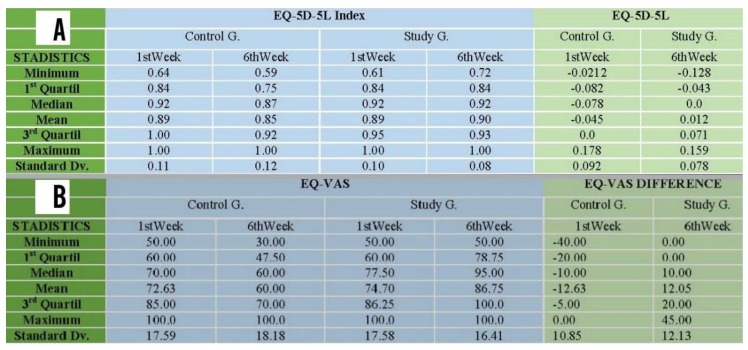
(**A**) Statistical description of the EQ-5D-5L Index and EQ-5D-5L Index differences for control and study groups. (**B**) Statistical description of EQ-VAS and EQ-VAS differences for control and study groups.

It was noted that the general scores obtained from the EQ-5D-5L Index for the control group decreased by the end of the 6 weeks (Figure 7A).

From the control group only 37% (seven participants) improved or maintained the EQ-5D-5L Index after 6 weeks. In contrast, from the study group, 68 % (14 participants) improved or maintained the EQ-5D-5L Index after 6 weeks.

**Figure 8:**
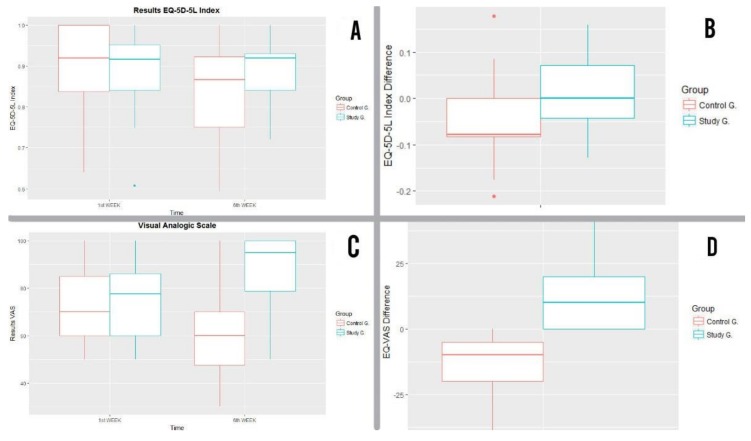
(**A**) Results obtained from the EQ-5D-5L Index in week 1 and week 6 for control and study groups. (**B**) Results of EQ-5D-5L Index differences in week 1 and week 6 for control and study groups. (**C**) Results of EQ-VAS in week 1 and week 6 for control and study groups. (**D**) Result of EQ-5D-5L Index differences in week 1 and week 6 for control and study groups.

In Figure 8A we can see that the EQ-5D-5L Index for the study group remained relatively stable with the median remaining at 0.92, whereas it decreased from 092 to 9.87 in the case of the control group (Figure 8A).

When analysing the difference in results in each group after 6 weeks, the mean EQ-5D-5L Index for the control group was less by 0.045, whereas in the study group the EQ-5D-5L Index produced a more stable mean (the mean of the differences was +0.012). (Figure 7A).

When comparing these changes using the t-test, the following null hypothesis was rejected: the treatment does not affect changes in EQ-5D-5L Index, with a 95% confidence level and value of *p* = 0.046 (Figure 8B). In accordance with the Wilcoxon test, the following null hypothesis was also rejected: there was no difference in improvement in health between the two groups being treated, with *p* value = 0.0391. (Figure 8B). In conclusion, in terms of the EQ-5D-5L Index, the study group remained stable whereas the control group slightly worsened. 

#### 3.4.2. EQ-VAS

Descriptively, an improvement was quickly able to be noted in EQ-VAS results for the study group. The mean increased from 74.7 to 86.8 and the range between the first and second quartile increased after 6 weeks from 60 and 86.25 to 78.75 and 100, respectively. Conversely, the mean for the control group decreased from 72.63 to 60 points, while the range between the first and second quartile also decreased after 6 weeks from 60 and 85 to 47.50 and 70 respectively (Figure 7B).

Visually, in Figure 8C the improvement of the study group compared with the control group was verified. The median of the study group went from 77.5 to 95, whereas in the control group it decreased from 70 to 60 from week 1 to week Figure 6. When analysing the difference in results in each group after 6 weeks, the mean EQ-VAS for the control group decreased (−12.63), whereas the EQ-VAS mean for the study group increased (12.05) (Figure 7B).

In accordance with the Wilcoxon test, we are rejecting the null hypothesis with a value *p* < 0.001 in favour of an alternative hypothesis that there is a difference in improvement in EQ/VAS results between the two groups being treated (Figure 8D).

In conclusion, in terms of the EQ-VAS, the control group worsened whereas the study group significantly improved.

### 3.5. Barthel Index

**Figure 9:**
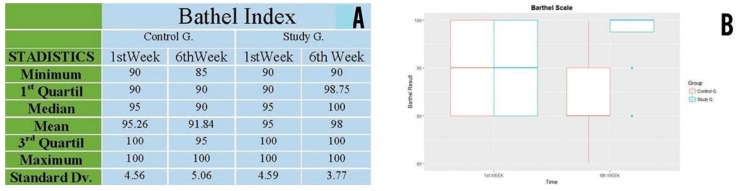
(**A**) Statistical description of the Barthel Index for control and study groups. (**B**) Results of Barthel Index In week 1 and week 6 for control and study groups.

Both control and study groups commenced the physical activity with homogenous features (minimum, maximum, mean and median) (Figure 9A).

After 6 weeks, the Barthel Index significantly improved in patients belonging to the study group. 50% attained the maximum score of 100 points whereas, conversely, the results obtained by patients from the control group substantially worsened. 

The results obtained from the Barthel Index in the study group improved after 6 weeks with statistically significant evidence, with a value of *p* < 0.003906. 

In contrast, the results obtained from the Barthel Index worsened in the control group after 6 weeks with statistically significant evidence, with a value of *p* < 0.001952. (Figure 9B).

### 3.6. Biofeedback: Physiological Constants

#### 3.6.1. Heart Rate

**Figure 10:**
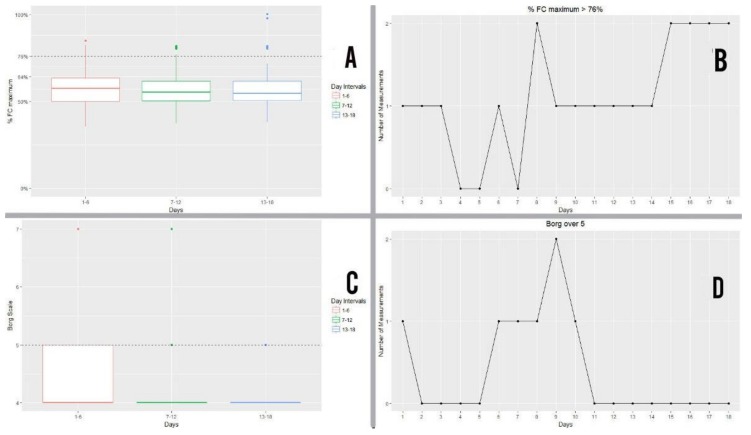
(**A**) Results of the maximum heart rate percentage for the study group during exercise, grouped together into 6-day intervals. (**B**) Number of % heart rate measurements (% FCmaximun) which were above the cut-off point per day. (**C**) Results of the intensity of perceived exertion according to the Borg scale for the study group during exercise, grouped together into 6-day intervals. (**D**) Number of perceived exertion measurements (Borg) which were above the cut-off point per day.

Below is the graph showing interquartile ranges of work intensity attained by patients from the study group during exercise, grouped together in 6-day intervals and expressed as a percentage of maximum heart rate (%HRMAX). Values below the broken line (76%) represented moderate, light levels of exercise intensity, meaning that patients below this line remained within suitable parameters of cardiac healthy and safe physical exercise. (Figure 10A)

Of the 360 heart rate measurements (20 patients x 18 days’ measurement) taken over the 18 days of exercise, 20 measurements failed to reach the cut-off point, i.e. only 5.55% of measurements were not below the 76% intensity cut-off point (Figure 10B). 94.5% of measurements remained below the cut-off point (Figure 10B).

#### 3.6.2. Modified Borg Scale

Below is the graph showing interquartile ranges of perceived exertion intensity attained by patients from the study group during exercise, grouped together in 6-day intervals and expressed according to the modified Borg scale. Values below the broken line (76%) represented moderate, light levels of exercise intensity, meaning that patients below this line remained within suitable parameters of cardiac healthy and safe physical exercise (Figure 10C

Of the 360 Borg scale measurements (20 patients x 18 days’ measurement) taken over the 18 days of exercise, seven measurements failed to reach cut-off point 5. In other words, 98.1% of measurements remained below the cut-off point (Figure 10D). Participants remained below the cut-off point except for two participants. (Figure 10D).

#### 3.6.3. Systolic Blood Pressure

**Figure 11:**
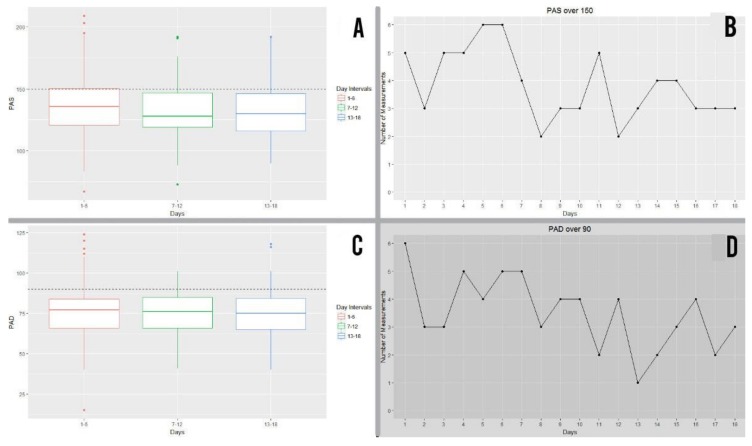
(**A**) Results of systolic blood pressure (SBP) for the study group during exercise, grouped together into 6-day intervals. (**B**) Number of systolic blood pressure (SBP) measurements which were above the cut-off point per day. (**C**) Results of diastolic blood pressure (DBP) for the study group during exercise, grouped together into 6-day intervals. (**D**) Number of diastolic blood pressure (DBP) measurements which were above the cut-off point per day.

Above is the graph showing interquartile ranges of systolic blood pressure (SBP) attained by patients from the study group during exercise, grouped together in 6-day intervals and expressed in millimetres of mercury (mmHg). Values below the broken line (150 mmHg) represented suitable levels of systolic blood pressure, meaning that patients below this line remained within suitable parameters of cardiac healthy and safe physical exercise. 75% of participants remained below the 150 line in the three intervals (Figure 11A). 

Of the 360 SBP measurements (20 patients x 18 days’ measurement) taken over the 18 days of exercise, 69 measurements (19.2%) exceeded the cut-off point, whereas 80% remained below the 150 cut-off point (Figure 11B). It was noted that the five participants who exceeded the cut-off point on the first day decreased to three participants by day 18 (Figure 11B).

#### 3.6.4. Diastolic Blood Pressure

Also above is the graph showing interquartile ranges of diastolic blood pressure (DBP) attained by patients from the study group during exercise, grouped together in 6-day intervals and expressed in millimetres of mercury (mmHg). Values below the broken line (90mmHg) represented suitable levels of diastolic blood pressure, meaning that patients below this line remained within suitable parameters of cardiac healthy and safe physical exercise. 75% of participants remained below the 90 line in the three intervals (Figure 11C Of the 360 DBP measurements (20 patients × 18 days’ measurement) taken over the 18 days of exercise, 63 measurements (17.25%) exceeded the cut-off point, whereas 82.5% remained below the 90 cut-off point (Figure 11D). It was noted that the six participants who exceeded the cut-off point on the first day decreased to three participants by day 18 (Figure 11D).

#### 3.6.5. Blood Oxygen Saturation (Sp0_2_)

**Figure 12:**
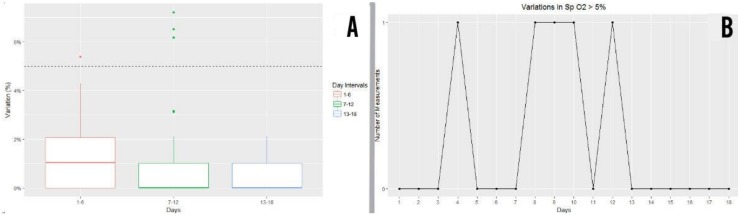
(**A**)Results of daily variations in blood oxygen saturation (SpO2) for the study group during exercise, grouped together into 6-day intervals. (**B**) Number of blood oxygen saturation measurements which were above the cut-off point per day.

Below is the graph showing interquartile ranges of daily variations in blood oxygen saturation (SpO2) attained by patients from the study group during exercise, grouped together in 6-day intervals and expressed as a percentage. Values below the broken line (5% SpO2) represented suitable levels of blood oxygen saturation, meaning that patients below this line remained within suitable parameters of cardiac healthy and safe physical exercise. In the last interval of the study, all patients were found to be with an SpO2 variation below 5% (Figure 12A).

Of the 360 SpO2 measurements (20 patients x 18 days’ measurement) taken over the 18 days of exercise, five measurements failed to reach the cut-off line (variation <5% SpO2) (Figure 12B). Therefore, only 1.4% of measurements were below the 5% cut-off point, whereas 98.6% of measurements remained at variation levels of <5% SpO2 (Figure 12B).

#### 3.6.6. Compliance with Suitable Parameters to Ensure that Physical Exercise is Cardiac Healthy and Safe 

**Figure 13:**
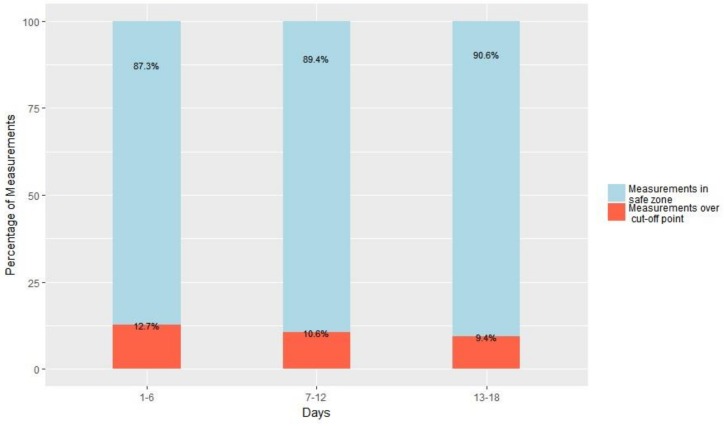
Measurement percentage within the parameters established as being safe for the following physiological constants grouped together into 6-day intervals.

As was described in the system’s design, the aim was for the proposed activity with the FRED game was not to entail any risk to the participant who carries it out. To this end and taking into account the parameters described in Figure 1 together with the maximum published scores for systolic and diastolic blood pressure and blood oxygen saturation [26,27,28], the percentage resulting from the sum of all physiological constant measurements was ascertained, namely: maximum heart rate, systolic blood pressure, diastolic blood pressure and blood oxygen saturation, taken from individuals belonging to the study group during exercise, grouped together into 6-day intervals and expressed as a percentage. Thus, compliance with suitable parameters may ensure that physical exercise be both cardiac healthy and safe. 1440 measurements were taken (four physiological constants x 20 participants x 18 days). Safety compliance of the exercise exceeded 87% in the three intervals and improved even more so as the days passed (Figure 13).

Attention should be drawn to the fact that in no case was the activity abandoned due to physical discomfort.

### 3.7. Software Usability Scale (SUS)

**Figure 14:**
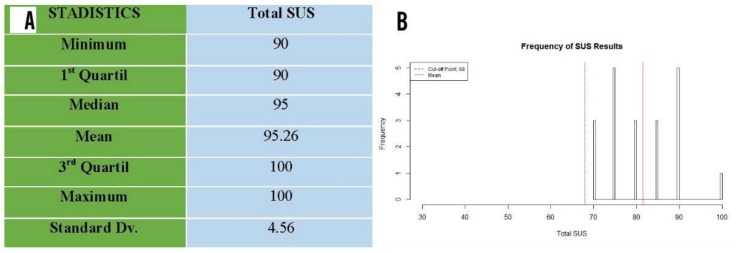
(**A**) Statistical description of the software usability scale (SUS) for study group. Figure 14B. Histogram of results obtained using the software usability scale (SUS); red line: mean; blue line represents cut-off point. (**B**) Histogram of results obtained using the software usability scale (SUS); red line: mean; blue line represents cut-off point.

With the range being considered from 0−100 for software usability scale (SUS) results, in which scores above 68 were deemed to be positive. It was noted that all participants who carried out the activity over 6 weeks using the FRED scored above the cut-off point (68) (Figure 14B). The lowest score obtained was 70 while the highest was 100 (Figure 14B). The mean was 81.5. This score indicates a good result in the SUS questionnaire (Figure 14A). The results showed major acceptance in terms of usability of the FRED game among participants from the study group.

### 3.8. Satisfaction with, Adherence to and Compliance with the FRED Game

Each participant from the study group was asked 2 questions following completion of the game each day, with a YES / NO response:

(1) Do you like the game?
(2) Do you find it motivating for the purpose of improving your physical condition?

**Figure 15:**
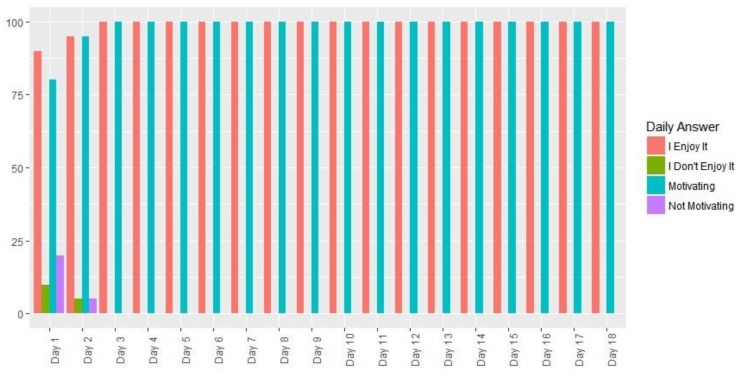
Daily response percentages by the study group to the questions: “Do you like the game?” and Do you find it motivating for the purpose of improving your physical condition?”.

In response to the first question, the 20 participants from the study group responded YES every day except days 1 and 2, in which case there was a negative response (10%―2 participants―and 5%―1 participant―respectively) (Figure 15).

In response to the second question, the 20 participants from the study group responded YES every day except days 1 and 2, in which case there was a negative response (20%―4 participants―and 5%―1 participant―respectively) (Figure 15).

## 4. Discussion

Our findings back up the hypothesis that FRED, an ad hoc-designed exergame, reduced the presence and severity of risk of frailty in a sample of sedentary elderly individuals to a significant extent, hence potentially altering their risk profile. The results we obtained, which are in keeping with previous evidence, suggest that elderly individuals with higher risk profiles may still benefit from preventive strategies and should not necessarily be excluded from any type of intervention that may help prevent disability.

The results are discussed in this section by contrasting them with those of other authors who have worked in similar, comparable areas of study. Cesari et al. [33] conducted a study in which the physical activity programme they arranged included aerobics (walking), tests of strength, flexibility and balance training, all of which were performed in three phases over a 12-month period: adoption (weeks 1−8), transition period (weeks 9−24) and maintenance (week 25 to month 12). The results showed that regular physical activity may help reduce frailty, especially among individuals deemed to be at higher risk of disability. 

Giné-Garriga et al. [34] conducted a study that combined functional balance and lower-body strength-based exercises over a 12-week period, known as the functional circuit training program (FCT). This entailed balance training 1 day a week, followed by lower-body strength-based exercises of 15 minutes’ duration each on another day. The results obtained showed that the functional circuit training program (FCT) can be effective in improving self-reported aspects including fear of falling and the general state of health in a group of physically frail individuals.

Clegg et al. [35] compared the effectiveness of the HOPE programme with usual care using a blind pilot randomised controlled trial (RCT), conducted over a 12-month period. This involved completing exercises of 15 minutes’ duration three times a day over 5 days of the week, with results showing that the trial provides initial evidence of the fact that a decline in mobility experienced by frail elderly individuals may be reduced via a 12-week exercise programme. Apart from mobility, independence in activities of daily living was also assessed via the Barthel Index, in which no significant differences between both groups was evidenced. As regards self-assessment of one’s state of health and quality of life via the EuroQol 5D-5L (EQ-5D-5L) questionnaire, the results showed a slight worsening. No data was provided about self-assessment of one’s state of health via the EuroQol-visual analogue scale (EQ-VAS).

Fairhall et al. [36] made a comparison between study group and control group with frail elderly individuals by setting in motion a multi-factor intervention programme with online exercises over a 12-month period. The results showed that the prevalence of frailty was 14.7% less in the intervention group than in the control group after 12 months. There were no significant differences between groups in terms of scores regarding the usefulness of the EuroQol 5D-5L (EQ-5D-5L) questionnaire. There was not any data about self-assessment of one’s state of health via the EuroQol-visual analogue scale (EQ-VAS).

Milte et al. [37] conducted a study over 2 weeks in order to explore the relative importance given to health and quality of life by elderly individuals who performed therapeutic gymnastics and undertook hydrotherapy sessions. Only results for the EuroQol 5D-5L (EQ-5D-5L) questionnaire were shown in this study, and no data was provided about the EuroQol-visual analogue scale (EQ-VAS).

Bieryla KA [38] used the Xbox Kinect™ to improve balance in elderly individuals, The results showed an improvement in terms of the balance tests carried out. However, no improvements were evidenced in the Timed Up and Go (TUG) test, which the author did not expect given that use of the Kinect™ compels participants to move more than other game systems.

The above studies [34,35,36,37,38] combine a range of physical activities which are of longer duration and performed at greater weekly frequency than the one carried out in our study using the FRED game. Although they manage to reduce frailty, they do so requiring far more time, and none of them contemplates exercise using an exergame. Only in the thee previous ones is reference made to the EuroQol 5D-5L (EQ-5D-5L) questionnaire, in which no significant differences are shown, whereas with the FRED game, the study group remained stable after 6 weeks while the control group slightly worsened. Unlike the other two authors referred to above, the EuroQol-visual analogue scale (EQ-VAS) was recorded in the study, in which the control group worsened whereas the study group significantly improved. Furthermore, the degree of independence for activities of daily living also evidenced an improvement after 6 weeks carrying out physical activity using the FRED game. Therefore, the degree of frailty is able to be reduced in less time using the FRED game, while the perception of one’s state of health and degree of independence in activities of daily living is much greater.

The study conducted by Daniel Daniel et al. [39] uses the exergame as a physical activity that can be performed to help try and limit frailty. In this case, the Nintendo^®^ and Wii™ consoles are used in conjunction with commonly-played games like bowling, tennis and boxing, which have been designed with the general public in mind, and these are then compared to seated exercise and control. Exercise sessions of 45 minutes’ duration were conducted three times a week over a 15-week period, and results pointed to the fact that all the differences indicated an improved physical functional state in the case of the seated exercise or Wii-fit groups, when compared to the control group. The FRED game differs from the above study because it managed to reduce the degree of frailty to a fifth of the time (3 weeks as opposed to 15), including sessions of less than half the duration (20 min as opposed to 45 min). In addition, the FRED game continued to reduce the degree of frailty over the 6-week period and consequently, it would seem to be more effective. 

The study conducted by Van Diest et al. [40] used the exergame to undertake unsupervised balance training at home, which involved the participant playing the role of a virtual ice skater for 30 minutes three times a week, over a six-week period. The pilot study ascertained on the one hand the fact that unsupervised home-based exergaming is a viable option in community-dwelling older adults, but also that participants do not necessarily gain the same benefit from such a programme, thus highlighting the need for more bespoke exergame training programmes. The conclusion drawn from this study clearly stresses the ad hoc nature of the FRED game when it comes to designing and preparing exercises, placing importance on the types of scenario to ensure they are creative and intuitive and capture the participant’s attention and interest. The specific movements that were carefully chosen for each scenario, respecting biomechanics, neuromotor functions and participation both of upper and lower extremities and the trunk, were able to facilitate independence in terms of basic activities of daily living (ADLs) such as transferring, dressing, eating, toileting and walking.

As regards physiological constants such as heart rate, systolic blood pressure, diastolic blood pressure and blood oxygen saturation, there are no studies in the literature reviewed that have measured these constants in elderly individuals in order to ascertain whether the physical exercise involved lies within certain safety parameters and whether it is in turn cardiac healthy. 

Scheer et al. [41] evaluated the effects of competing against a computer or human opponent in young adults on a person’s heart rate, ventilation, oxygen consumption and amount of energy expended using Nintendo Wii™, Sony Move and Microsoft Kinect™ games. The results obtained in the case of oxygen consumption were then used to compare Nintendo Wii, Sony Move and Microsoft Kinect™ games by taking into account moderate health guidelines suggested to encourage physical activity.

In their study, O’Donovan et al. [42] compared the amount of energy expended when playing in both individual and multi-player mode using Xbox Kinect™ and Wii™ consoles. They concluded that the amount of energy expended together with the person’s heart rate increased when playing with the Xbox Kinect™ console in multi-player mode.

There is a recent publication by Barry el al. [43], in which they describe the study conducted to compare the effects of exergaming with traditional gymnastics-based exercises in which patterns of movement, intensity and physiological demand were similar for postural control. All participants (young adults) completed three exercise sessions of 30 minutes’ duration a week over a 4-week period. Heart rate was compared between groups and no differences were found. Ratings for perceived exertion (RPE) or the Borg scale were significantly lower in the Kinect ™ group. The Kinect ™ group perceived less physical exertion than the group which carried out traditional gymnastics-based exercises. There were also significant differences in terms of acceptance of the technology between groups and in considering it to be a positive exercise experience, with higher scores in the case of the Kinect exercise group. They concluded that exergaming with Kinect ™ may attain moderate levels of physical exercise intensity with positive feelings about the reduction in perceived exertion in comparison to traditional types of exercise. 

In this sense, we obtained using similar results in terms of the fact that a moderate level of physical activity intensity may be obtained, accompanied by a low rating of perceived exertion. This was also accepted positively and proved a motivating force in improving the individual’s physical condition. 

Karahan et al. [44] conducted a study to compare the effects between exergames using the Xbox Kinect™ device and exercise at home involving balance training, functional mobility and lity of life among individuals over 65 years of age. Each participant played for 30 minutes 5 days per week over a 6-week period (30 sessions in total) in the company of an experienced nurse, who was able to carry out cardiopulmonary monitoring in order to ensure there were no events and, thus, ensure the safety of the exercise being undertaken. The extent to which participants enjoyed it was in turn assessed using a 5-point Likert scale (0−5). Results were positive and both groups improved in terms of balance, functional mobility and quality of life, although the authors pointed out that the group which did the exercise using the Xbox Kinect™ device obtained better results. 

In this sense, our results differ because it resulted in a major difference in terms of physical condition after just three sessions a week and for less duration, as the risk of frailty evidenced in the study group was significantly reduced whereas it considerably worsened in the control group. Similarly, it did so in terms of independence in activities of daily living, with the study group obtaining better results in the Barthel Index than the control group, which obtained a worse result after 6 weeks. As regards compliance with safety with a view to preventing any event from taking place that might put the participants at risk, physiological constants were measured in the FRED game such as heart rate, rating for perceived exertion, systolic blood pressure, diastolic blood pressure and blood oxygen saturation. These measurements were taken before commencing, immediately afterwards and 5 minutes after having completed in order to make an assessment taking into account the safety parameters established and published by European guides, resulting in a cardiac healthy and safe exercise because safety compliance of the physical exercise exceeded 87% and even improved as the days went by. Moreover, in no case was the activity abandoned due to physical discomfort. 

A recent publication by Meneghini et al. [45] involves a qualitative study about the perception of elderly people with regard to participation in exergaming-based exercise. Fourteen individuals (55−77 years of age) carried out 12 weeks of exercise (50 min, 3 days/week) using Xbox 360 Kinect™ Sports. Participants reported psychological benefits (self-esteem, concentration, mood, reasoning, memory and wellbeing), physical benefits (agility and physical conditions) and social interaction (exchange of experiences, friendship and competitiveness). As regards group experiences, innovation, the game itself and visual stimulation were cited as features of the games, and the perception of benefits from participation in exergames fostered the pursuit of exercise and increases motivation among participants. In this sense, in the case of the FRED game, it managed to reduce the frailty risk that benefit from physical exercise entails by half the time (weeks) and in sessions of less than half the duration. It also managed to improve the degree of independence in activities of daily living and in the perception of participants’ state of health. The study group awarded the game very high scores, meaning that the game was accepted and rated very positively in terms of its usability. The game was also adhered to and complied with by the study group, as all and every one of the participants completed the same number of sessions and responded 100% to the daily questions to ascertain whether they liked the game and whether they found it motivating for the purpose of improving their physical condition. All the benefits obtained and described previously are due to the added appeal offered by the FRED game over other types of existing game. This appeal of being an ad hoc designed game in which the idea is for the user to be the protagonist achieves greater involvement in, adherence to and compliance with physical exercise.

## 5. Conclusions

The ad hoc developed FRED game helped to reduce the degree of frailty by improving the functional capacity of frail elderly individuals, thus enabling them to remain independent as long as possible. 

Frail elderly individuals improved the perception of their state of health and quality of life while in turn their mood was improved, they became motivated towards participation and integration and, hence, were able to prevent the sense of isolation which they would otherwise easily tend towards.

The FRED game helped to maintain and improve independence in activities of daily living in frail elderly individuals. 

The FRED game showed that it is able to motivate frail elderly individuals in doing exercise, because it involved a game that they liked and which proved motivating for the purpose of improving their physical condition. The feature of having been designed ad hoc proved very appealing among frail elderly individuals, who felt themselves to be the protagonists and thus achieved better involvement in, adherence to and compliance with physical exercise. The activity proposed with the FRED game did not entail any risk to the frail elderly individuals who took part in it, given that they worked within safe and cardiac healthy parameters. 

The study undertaken confirms the fact that the FRED game proves to be a valid technological solution for reducing frailty risk. 

Based on the study conducted, the exergame may be considered to be an effective, safe and entertaining alternative with which an improvement is made not only in terms of the physical and functional capacity of the individual, but also an improvement in psychological and social aspects which, in short, make the individual evidence a greater degree of independence over a longer time, i.e. “bring more life to the years”. 

The possibility of carrying out a study in the near future is under consideration, in which prevention of the onset of frailty could be studied. 

Limitations

The biggest limitation in the study was the sample size. On the other hand, the participation of a greater number of day centers would help to enlarge the sample, but they only accepted 2 centers. For these reasons, the authors would like to carry out future research on a larger scale. 

## Abbreviations

EQ 5D-5L	EuroQol 5 dimensions-5 levels
EQ-VAS	EuroQol Visual analogue scale
SPPB	Short Physical Performance Battery
SUS	System usability scale
MET	Metabolic equivalent
RPE	Rating of perceived exertion
HR	Heart Rate
%HRMAX	Percentage of maximum heart rate
SBP	Systolic blood pressure
DPB	Diastolic blood pressure
SpO2	Blood oxygen saturation
TV	Television
3D	Three-dimensional
CONSORT	Consolidated Standards of Reporting Trials
